# Simultaneous extraction of proteins and metabolites from cells in culture

**DOI:** 10.1016/j.mex.2014.07.002

**Published:** 2014-07-18

**Authors:** Sean C. Sapcariu, Tamara Kanashova, Daniel Weindl, Jenny Ghelfi, Gunnar Dittmar, Karsten Hiller

**Affiliations:** a Luxembourg Centre for Systems Biomedicine, University of Luxembourg, L-4362 Esch-Belval, Luxembourg; b Mass Spectrometry Core Unit, Max-Delbrück Center for Molecular Medicine, 13125 Berlin, Germany; c HICE – Helmholtz Virtual Institute of Complex Molecular Systems in Environmental Health – Aerosols and Health, Germany^1^1 www.hice-vi.eu.

**Keywords:** Sample preparation, Metabolomics, Proteomics, Biomolecule extraction, Isolation, GC/MS, LC/MS

## Abstract

Proper sample preparation is an integral part of all omics approaches, and can drastically impact the results of a wide number of analyses. As metabolomics and proteomics research approaches often yield complementary information, it is desirable to have a sample preparation procedure which can yield information for both types of analyses from the same cell population. This protocol explains a method for the separation and isolation of metabolites and proteins from the same biological sample, in order for downstream use in metabolomics and proteomics analyses simultaneously. In this way, two different levels of biological regulation can be studied in a single sample, minimizing the variance that would result from multiple experiments. This protocol can be used with both adherent and suspension cell cultures, and the extraction of metabolites from cellular medium is also detailed, so that cellular uptake and secretion of metabolites can be quantified.

Advantages of this technique includes:

1. Inexpensive and quick to perform; this method does not require any kits.
2. Can be used on any cells in culture, including cell lines and primary cells extracted from living organisms.
3. A wide variety of different analysis techniques can be used, adding additional value to metabolomics data analyzed from a sample; this is of high value in experimental systems biology.

## Graphical abstract

**Figure:**
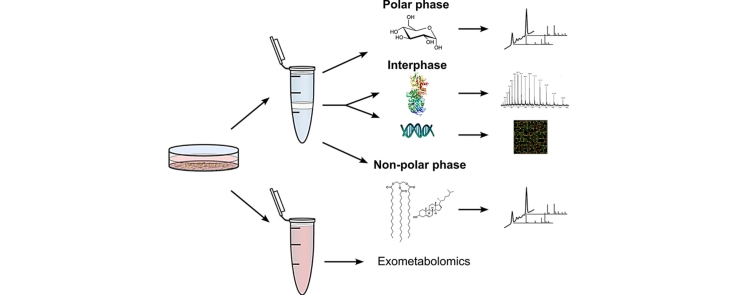

Three-phase methanol–water–chloroform extraction for biological samples. Examples of components available from each phase are shown. These different phases can be then used for a variety of different analysis methods on different levels of cellular regulation.

## Method details

### Washing cells, quenching metabolism, extraction and separation of phases

#### Materials

• Living cells, cultured on petri dishes or multi-well plates. Cells can be incubated with a labeled tracer (for example: ^13^C or ^15^N) for downstream flux analysis
  – Cells should be confluent in the wells, with a consistent cell number between samples. The amount of cells will vary depending on cell type used, but we have found using around 1 million cells in each well of a 6-well multiwell plate gives good results for both techniques.
• 0.9% NaCl at room temperature
• High purity (MS grade) methanol at −20 °C
• High purity (MS grade) chloroform at −20 °C
• Millipore or equivalently pure water on ice
• Cell scrapers
• Eppendorf tube shaker at 4 °C
• Centrifuge at 4 °C
• *Note*: This list does not include generic laboratory equipment, which are assumed to be available.

The metabolic profile of a cell can change in as little as a few seconds. Therefore, the most important step in metabolite extraction is the quenching of metabolism; this ensures that the metabolic pathways in the cells do not continue to function, and that the cellular state at the point of extraction is as close as possible to the desired analysis time point [1]. This quenching must be performed quickly. There has been much discussion as to which extraction fluids are best for quenching and measuring metabolites [2,3]; however, it is generally agreed that a mixture of water and methanol provides the best extraction efficiency with minimal loss. Both fluids are added directly to the cells, and should be kept as cold as possible (methanol at −20 °C and water on ice).

Once the metabolic processes have been quenched, the next step is to lyse the cells, separating both the polar and non-polar metabolites from the other cellular substances at the same time. While methanol and water will extract the polar metabolites from a sample, non-polar metabolites must be separated with a non-polar solvent. Therefore, we use chloroform [4] with the methanol/water mixture to separate the polar and non-polar metabolites efficiently. Adherent cells quenched with methanol and water are scraped from the multi-well plates and added to cold chloroform to allow for separation of polar and non-polar phases. These extracts are agitated to complete cell lysis and centrifuged to fully separate the layers.

This is a crucial step for experimental consistency; different amounts of cells in different samples will lead to incorrect comparisons of metabolite levels (which can also occur with cell seeding). Therefore, care should be taken to adequately scrape all wells and transfer as much cellular material as possible from the wells to the chloroform.

After these steps, the cells are shaken to completely lyse the membranes allowing for a more efficient extraction of all possible biomolecules. After shaking, there should be a clear separation between the polar and non-polar phase for the metabolites, with a well-defined interphase containing proteins and nucleic acids.

#### Procedure – adherent cells

The following procedure is for adherent cells cultured on a 6-well multiplate. For cells cultured on a 12-well multiplate, divide the amount of extraction fluids by two. Amounts for other cell culture vessels should be adjusted accordingly.

1. Retain medium for quantification of cellular consumption and secretion of metabolites.
2. Wash cells with 1 mL 0.9% NaCl. Phosphate buffer solution is avoided because it would create a large phosphate peak during mass spectrometric analysis, masking lower intensity metabolites.
3. Quench cells by adding 400 μL methanol at −20 °C followed by 400 μL Millipore H_2_O on ice. As soon as the methanol has been added, place the multiwell plate on ice for the next steps.
4. Scrape wells with a cell scraper. Make sure that as few cells as possible remain attached to the plate through careful and thorough scraping.
5. Transfer cell extract into an Eppendorf tube containing 400 μL chloroform at −20 °C.
6. Agitate cells in a tube shaker (pre-cooled to 4 °C) for 20 min at 1400 rpm, followed by 5 min of centrifugation at a minimum of 16,100 × *g* at 4 °C.

#### Procedure – suspension cells

The following procedure is for suspension cells cultured on a 6-well multiplate. For cells cultured on a 12-well multiplate, divide the amount of extraction fluids by two. Amounts for other cell culture vessels should be adjusted accordingly.

1. Centrifuge cells in medium at 250 × *g* for 5 min to pellet the cells, collect medium and retain for quantification of cellular consumption and secretion of metabolites.
2. Wash cells with 1 mL 0.9% NaCl, centrifuge at 250 × *g* for 5 min to pellet the cells, then discard NaCl.
3. Quench cells by adding 400 μL methanol at −20 °C and 400 μL Millipore H_2_O on ice.
4. Add 400 μL chloroform at −20 °C to the cells, transfer to Eppendorf tubes.
5. Agitate cells in a tube shaker (pre-cooled to 4 °C) for 20 min at 1400 rpm, followed by 5 min of centrifugation at a minimum of 16,100 × *g* at 4 °C.

#### Procedure – cell types with both adherent and suspension fractions

For cell types which are both suspended and adherent in culture, the procedure must be modified somewhat to avoid over-dilution of the metabolites. The following procedure is for cells cultured on a 6-well multiplate. Amounts for other cell culture vessels should be adjusted accordingly.

1. Collect the medium from the plate, centrifuge cells in medium at 250 × *g* for 5 min to pellet the cells, collect medium and retain for quantification of cellular consumption and secretion of metabolites.
2. Wash the adherent fraction and suspension fraction of cells each with 1 mL 0.9% NaCl, centrifuge at 250 × *g* for 5 min to pellet the cells, then discard NaCl.
3. Quench cells by adding 200 μL methanol at −20 °C and 200 μL Millipore H_2_O on ice to both the suspension cell pellet and the adherent cell pellet. As soon as the methanol has been added, place the multiwell plate on ice for the next steps.
4. Scrape wells containing adherent cell fraction with a cell scraper. Make sure that, as few cells as possible remain attached to the plate through careful and thorough scraping. Transfer the quenched cells to the corresponding non-adherent cell fraction.
5. Add 400 μL chloroform at −20 °C to the cells, transfer to Eppendorf tubes.
6. Agitate cells in a tube shaker (pre-cooled to 4 °C) for 20 min at 1400 rpm, followed by 5 min of centrifugation at a minimum of 16,100 × *g* at 4 °C.

### Separation of phases and polar metabolite extraction

#### Materials

• Glass vials for GC analysis
• Rotary vacuum evaporator
• High purity (MS grade) methanol at −20 °C

After centrifugation, the different phases are now separated, and each one can be sampled for further analysis with multiple techniques. For gas chromatography coupled to mass spectrometry (GC/MS) analysis, samples need to be transfered to a glass vial and dried at a low temperature under vacuum to avoid metabolite degradation. No liquid should remain in the glass vials after drying, and vials should be brought to room temperature under vacuum to avoid condensation. If liquid is still present in the vial, simply dry further in the rotary evaporator. For liquid chromatography coupled to mass spectrometry (LC/MS) analysis, the polar phase can be used directly for injection after transferring to a glass vial.

#### Procedure

The following procedure is for adherent cells cultured on a 6-well multiplate. For cells cultured on a 12-well multiplate, divide the amount of polar phase extracted by half.

1. Carefully transfer 300 μL of the polar (upper) phase to a glass vial without touching the interphase.
2. Evaporate polar phase in a rotary vacuum evaporator at −4 °C until dry (or overnight). When vials are dry, raise temperature on the rotary vacuum evaporator to room temperature for 30 min before removing the vials to avoid condensation. After capping vials, store at −80 °C until analysis.
3. Remove unused polar phase while avoiding the removal of any interphase. After polar phase is almost completely removed, tilt the Eppendorf tube at a 45°, so that the interphase moves out of the way and the non-polar phase is more easily accessible. If analysis of non-polar phase is to be performed, transfer 300 μL into a glass vial and dry the same way as the polar phase. Otherwise, discard the non-polar phase, being careful not to remove the interphase.
4. Wash the interphase with 300 μL methanol at −20 °C, and centrifuge for 10 min at a minimum of 16,100 ×*g* at 4 °C.
5. Remove methanol, process interphase to extract proteins or mRNA for further analysis. Alternatively, add 50 μL methanol at −20 °C, and store interphase at −80 °C until further extraction of nucleic acids or proteins.
6. *Optional: For metabolomics analysis of amino acids in the protein fraction, hydrolyze the interphase as follows:* Remove methanol from interphase. Heat interphase overnight in 400 μL of 6M HCl in a tightly sealed tube at 100 °C. Transfer 100 μL to a glass vial and dry in a rotary vacuum evaporator at −4 °C until dry. Store at −80 °C until analysis.

### Protein extraction from interphase and sample preparation for LC/MS proteomics analysis

#### Materials

• Denaturation buffer (6 mol/L urea, 2 mol/L thiourea, 20 mmol/L HEPES, adjusted to pH 8.0)
• 10 μmol/L TCEP (tris(2-carboxyethyl)phosphine), dissolved in ABC-buffer (50 mmol/L ammonium bicarbonate, adjusted to pH 8.5)
• 55 mmol/L chloroacetamide, dissolved in ABC-buffer (as above)
• 0.5 μg/μL Endopeptidase LysC dissolved in ABC-buffer (as above) – trypsin or other proteases can also be used (at the same concentration)
• 10% trifluoroacetic acid (TFA)
• Rotary vacuum evaporator
• Sonicator
• Stage tips, used for desalting

For proteomics analyses, it is necessary to further process the interphase so that the proteins contained within can be analyzed on an LC-MS/MS instrument. For the proteomics analysis the proteins have to be chemically modified and digested to peptides. The proteins are first unfolded and the disulfide bonds are reduced, removing their tertiary and secondary structure, leaving only a chain of amino acids. The second step is the alkylation of the cysteines in order to prevent the spontaneous formation of disulfide bonds. The last step is the digest with a specific protease to generate peptides for the LC-MS/MS analysis. In case the LC system is not equipped with a pre-column the peptides can be concentrated and desalted using stage-tip purification [5].

#### Procedure

1. Dry the washed interphases (containing the protein fraction) for 15 min in a rotary vacuum evaporator at 35 °C.
2. Resuspend the proteins in 60 μL of denaturation buffer, and sonicate for 1 min to break up protein aggregates.
3. Quantify proteins using a Bradford assay, and isolate a total amount of 100 μg of protein.
4. Mix 2 μL of the TCEP solution with the 100 μg of the sample protein (in solution) in a new Eppendorf tube, and incubate for 30 min at room temperature to reduce the disulfide bonds in the proteins.
5. Add 1 μL of chloroacetamide solution to 10 μL of the sample protein, and incubate for 20 min at room temperature, alkylating the cysteine residues.
6. Add 4 μL of LysC to the solution, and incubate for 3 h at room temperature to digest the proteins. At this point, the samples can be acidified to pH <2.5 by adding 10 μL of 10% TFA solution to stop the digestion, fractionated, desalted using stage tips, and analyzed using LC-MS/MS.
  *As an alternative to the LysC digestion in step 6, the proteins can be digested using trypsin using the following steps:*
  • In this case, dilute the sample with 4× ABC (Make sure the end concentration of urea in the sample does not exceed 2 mol/L).
  • Next, add 2 μL trypsin and incubate overnight at room temperature. Stop the digestion by adding 10 μL of 10% TFA solution, acidifying the sample to pH <2.5. At this point, the samples can be fractionated, desalted using stage tips, and analyzed using LC-MS/MS.

### Extraction of metabolites from cell culture medium

#### Materials

• An 8:1 mixture of methanol:H_2_O at −20 °C
• Glass vials for GC analysis
• Centrifuge at 4 °C
• Refrigerated rotary vacuum evaporator

An important part of characterizing the metabolic state of cells is understanding the uptake and secretion of biomolecules. This can be measured through metabolomic analysis of cell culture medium used in an experiment. This information can be used to understand the energetic needs of a cell in different conditions, and is complementary to the amounts of metabolites inside cellular compartments. For analysis methods such as ^13^C-metabolic flux analysis, quantification of cellular uptake and secretion rates are a vital part of the information needed to infer metabolic fluxes [6].

#### Procedure

The following procedure is for high glucose medium (25 mmol/L). For media with less glucose, the dilutions can be reduced.

1. Prepare Eppendorf tubes with 450 μL 8:1 methanol:H_2_O at −20 °C.
2. Mix medium well; transfer 50 μL to the extraction fluid. Mix by shortly vortexing.
3. Centrifuge tubes for 5 min at a minimum of 16,100 × *g* at 4 °C.
4. Transfer 100 μL of supernatant to a glass vial and dry in a rotary vacuum evaporator at −4 °C. Cap and store vials at −80 °C until analysis.

### Optional: extension of this technique

If desired, alternate extraction techniques can be applied to the interphase after metabolic extraction. As the interphase contains both nucleic acids and proteins, these biomolecules can be isolated for alternate forms of analysis simultaneously with metabolomics. A hydrolysis of the interphase would break down the biomolecules, allowing analysis of individual nucleobases or amino acids using GC/MS and LC/MS. Nucleic acids, such as mRNA or DNA, can be extracted and used for transcriptomics and genomics analyses (microarrays, qPCRs, and others).

## Additional information

Integration of biological data across multiple levels of regulation increases the robustness of any experimental result. In many cases, separate experiments are performed (either sequentially or in parallel) using the same conditions, in order to target proteins, metabolites, RNA, or DNA through the individual experiments. Even though care is taken to minimize variation, differences between experiments can sometimes occur due to forces outside of the experimentalist's control. Methods which enable extraction of multiple types of biological material from single experiments allow direct comparison of different cellular activities.

Sample preparation determines the overall sensitivity, accuracy, and robustness of a biological analysis, and is therefore a very important step in experimental design. Thus, it is also the ideal place to modify a protocol for the extraction of multiple type of biological materials. Since Bligh and Dyer published their protocol on extraction of lipids [4], there has been a consistent effort to improve upon extraction methods for metabolomics analyses. Particularly in recent times, as the methods and techniques of metabolomics have increased in use, there has been a concurrent increase of comparisons of different improvements on metabolite extraction protocols.

Complementary to metabolomics analysis, mass spectrometry-based proteomics can be used to investigate the protein composition of a cell, to determine the members of protein complexes, their structure, the protein composition of organelles, and the dynamics of these processes. Protocols for the extraction of proteins have also been steadily improving over the last few decades [7,8], and it is possible to take advantage of the similarities between these techniques and metabolomics extraction techniques to extend current methods.

As it has been often stated that the chloroform/methanol/water extraction is optimal over a large range of compound classes [2,3,9], we intend to broaden the scope of this protocol, including a method for quantification of cellular metabolic uptake and secretion as well as adding the ability for simultaneous proteomics analysis (or analyses of other “omics” levels) in the same sample. This will allow for more robust systems biology approaches for the integration of different cellular regulatory levels, where metabolomics can be used as a base for the understanding of different cellular phenotypes.

It should be noted that this extraction technique could be applied for a wide variety of different approaches. Modern techniques, such as ^13^C-flux analysis, stable isotope labeling by amino acids in cell culture (SILAC) proteomics, or the analysis of the posttranslational modification state of proteins and their dynamics [8] can be used along with this protocol.

Roume et al. [10] have previously described a comprehensive method for extraction from microbial communities; while this method is comprehensive, it requires multiple kits and a large time commitment. The protocol described here is inexpensive and relatively quick to perform. In addition, our protocol is designed for and tested on mammalian as opposed to microbial cells. Weckwerth et al. [11] also have an existing protocol for extraction of multiple types of biomolecules from a single sample, but their protocol is designed primarily for plant cells. More steps are necessary to break down the plant cell walls, which increases the time and complexity compared to the technique described here.

## Conflicts of interest

The authors declare no conflicts of interest.
